# Exosomes Derived from Bone Marrow Dendritic Cells Exhibit Protective and Therapeutic Potential Against Chemically Induced Chronic Pancreatitis in Rats

**DOI:** 10.1007/s10753-024-02150-y

**Published:** 2024-10-19

**Authors:** Shaimaa M. Bashir, Sherine M. Rizk, Mohammed M. Nooh, Hebatullah S. Helmy

**Affiliations:** 1 Department of Biochemistry, Faculty of Pharmacy, October 6 University, Giza, 12585 Egypt; 2 Department of Biochemistry, Faculty of Pharmacy, Cairo University, Cairo, 11562 Egypt

**Keywords:** Chronic pancreatitis, Dendritic cells, Exosomes

## Abstract

**Background:**

Chronic pancreatitis (CP) is a specific clinical disorder that develops from pancreatic fibrosis and immune cell dysregulation. It has been proposed that bone marrow dendritic cells (BMDCs) exosomes have significant effects on immune regulation. Thus, the current study acquainted the prophylactic and therapeutic effects of exosomes derived from BMDCs on a rat model of CP.

**Materials and methods:**

BMDCs were prepared and identified, and then the exosomes were isolated by differential ultracentrifugation. Prophylactic and therapeutic effects of exosomes were investigated on L-arginine induced CP model.

**Results:**

Administration of two tail vein injections of exosomes (200 μg/kg/dose suspended in 0.2 ml PBS) markedly improved the pancreatic function and histology compared to CP group. Moreover, exosomes prominently mitigated the increase in amylase, lipase, tumor necrosis factor-α (TNF-α), transforming growth factor-β (TGF-β) and elevated antioxidant enzymes; catalase, superoxide dismutase (SOD) and glutathione peroxidase (GPx).

**Conclusion:**

BMDCs exosomes can be considered as a promising candidate, with a high efficacy and stability compared with its parent cell, for management of CP and similar inflammatory diseases.

## INTRODUCTION

Chronic pancreatitis (CP) is a specific, typically difficult to distinguish clinical disorder which develops from progressive pancreatic inflammation [1].The most common symptom of CP is severe abdominal pain which impacts seriously the quality of life of the patients [2]. Long-term complications include insufficiency of exocrine and endocrine pancreatic functions, diabetes mellitus, and ductal adenocarcinoma [3]. The reported annual incidence rates of CP are basically similar in all countries [4], ranging from 5 to 14 per 100,000 individuals, with a prevalence of roughly 30–50 per 100,000 individuals. CP has been found to be associated with a high mortality rate of approximately 50% within 20–25 years of diagnosis [5]. Additionally, CP is identified as the strongest risk factor for pancreatic cancer, increasing its occurrence probability by about 13.3 fold [6].

CP is most common in alcoholics, smokers, and people with metabolic abnormalities. Pancreatic insults initiate acute pancreatitis, which deregulates immune cells and recruits inflammatory cells. Further insults lead to recurrent episodes of acute pancreatitis, which trigger the activation of pancreatic stellate cells (PSCs) and initiate pancreatic fibrogenesis, ultimately causing CP which is characterized by immune cell infiltration and dysregulation of immune cells; mainly T cells [1]. The treatment of CP represents a big challenge for researchers due to the complex pathophysiology and vague etiology of this debilitating disease. Currently, supportive care via nutrition and pain management is the main treatment strategy for CP [7, 8].

Dendritic cells (DCs) are specific antigen-presenting cells that deliver essential signals to improve innate and adaptive immune responses. A sequence of maturation steps, including antigen uptake, antigen processing, and migration to specialized lymphoid organs, are directed by DCs in response to the detection of invading pathogens or other inflammatory stimuli. This response is characterized by substantial transcriptional and epigenetic reprogramming [9].

Exosomes are small membrane vesicles, of 40–150 nm, released into the extracellular fluids when endosomes fuse with the plasma cell membrane [10]. Exosomes contain a variety of materials, including proteins, lipids and nucleic acids such as mRNAs and micro-RNAs [11]. They are similar to their parent cells regarding the composition and characteristics. But unlike their parents, exosomes exhibit sustained biological activity and are less likely to be rejected by the immune system. Exosomes impact target cells, causing them to function differently [10]. The therapeutic efficacy of exosomes has been indicated in different disease cases, such as myocardial infarction, liver and kidney injury, hind limb ischemia and inflammatory diseases such as arthritis [12] and periodontitis [13]. Exosomes produced by DCs have T-cell co-stimulatory molecules and major histocompatibility complex classes I (MHC I) and II (MHC II) on their surface, indicating that they are involved in immune regulation process [14]. DCs-generated exosomes showed neuroprotective effect and mitigated the development of autoimmune encephalomyelitis in vitro, via regulating the balance between T helper cells and regulatory T cells [15]. To date, few studies explored the possible beneficial role of exosomes against CP. Therefore, the present study investigated the protective and therapeutic outcomes of DCs-derived exosomes in a rat model of CP via monitoring some biochemical and histological changes.

## MATERIALS AND METHODS

### Experimental Animals

The study was conducted on male Wistar albino rats (150–200 g) which were provided by the Egyptian organization for biological products and vaccines, Cairo, Egypt. Rats were bred under controlled humidity and temperature and 12 h light/dark cycle in a well-aerated animal house of Faculty of Pharmacy, October 6 University and were offered water and commercially available diet pellets throughout the study. Animal care and the experimental procedures adhered strictly to the standards of the Ethics Committee of the Faculty of Pharmacy, Cairo University, Cairo, Egypt (Permit number: BC 2402) and conformed to the principles of the Declaration of Helsinki.

### Preparation and Identification of BMDCs

#### BMDCs Generation

DCs were produced from bone marrow (BM) progenitor cells as previously described [16]. Briefly, BM cells were collected from tibias and femurs of five rats by using Roswell Park Memorial Institute (RPMI) medium (sigma-Aldrich, USA) to flush the marrow out of the bones. The media-cell mixture was centrifuged at 13500 xg for 5 min. The cell pellet was separated, to which ammonium chloride lysis buffer was added followed directly by ice-cold Phosphate-buffered saline (PBS) to dilute the buffer. PBS-cell mixture was then centrifuged at 13500 xg for 5 min to obtain the cell pellet which was purified by being suspended in ice-cold PBS and further centrifuged at the same speed for 5 min. Finally, the cell pellet was separated and resuspended in cold RPMI complete medium. 50 µL of cell suspension was withdrawn for viability and quantification analysis by using homocytometer on a Bright-field microscope and trypan dye (0.4%, w/v) [17]

#### BMDCs Culture

Cells were cultured in RPMI complete medium containing recombinant rat granulocyte–macrophage colony stimulating factor (rrGM-CSF), (biotechne, CAT. #: 518-GM), recombinant rat interleukin-4 (rrIL-4), (biotechne, CAT. #: 504-RL) and recombinant rat tumor necrosis factor-α (rrTNF-α), (biotechne, CAT.510-RT) (Table 1).

**Table 1:**
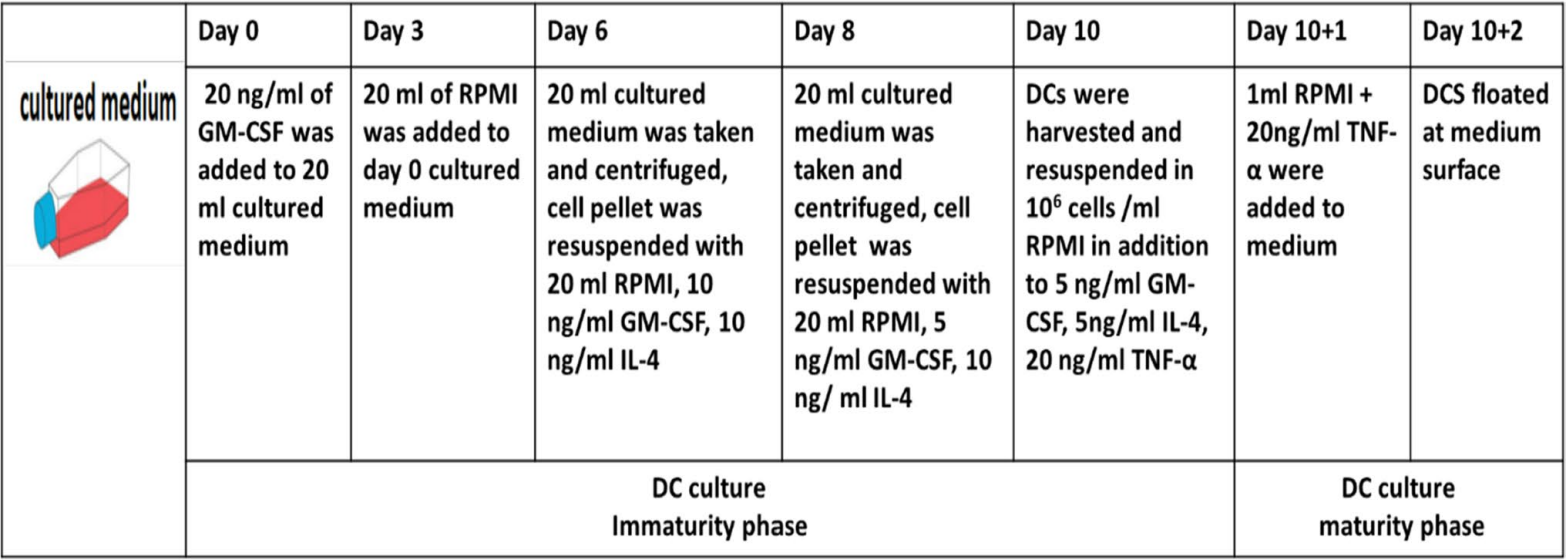
BMDCs Culture Protocol

#### Morphological Detection of DCs

Inverted phase-contrast microscope (Leica, Germany) was used to track the continuous morphological changes of DCs, while the structure of DC surface was imaged using an electron microscope ( HITACHI, H-7650, Japan) [18].

#### Flow Cytometric Detection of DC Phenotypes

Cell suspensions were prepared to each a different antibody was added for further analysis by flow cytometry using Fluorescence-labeled IgG isotypes as control [18]. The used antibodies were PE-labeled anti-CD11c (OriGene Technologies, USA, AM05861PU-S), PE-labeled anti-CD80 (abcam, UK, ab215166), PE-labeled anti-CD86 [BU63]—BSA and Azide free (abcam, UK, ab213044,), FITC-labeled anti-CD40 (SANTA CRUZ BIOTECHNOLOGY, INC, USA (H-10): sc-13128) and PE-labeled MHCII (SANTA CRUZ BIOTECHNOLOGY, INC, USA (ER-TR3): sc-59318).

### Purification and Identification of BMDCs Derived Exosomes

**Fig. 1:**
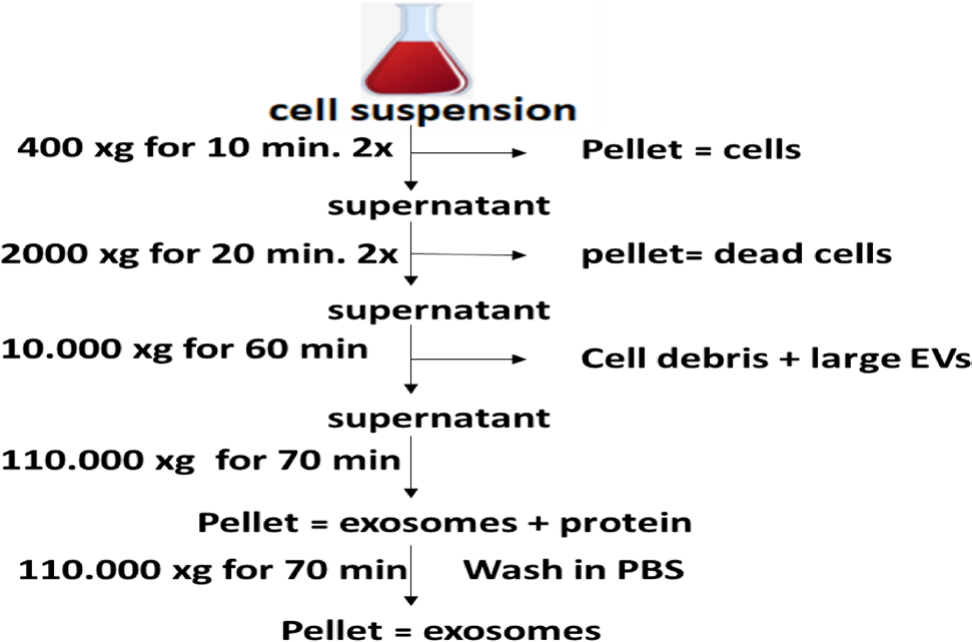
Flowchart of exosome purification protocol. Exosomes are isolated by differential centrifugation steps with increasing speeds from BMDCs suspensions and pelleted by ultracentrifugation.

As shown in Fig. 1, The cell suspension was subjected to seven consecutive centrifugation steps at progressively higher speeds. At each of the first five steps the pellet was discarded and the supernatant was subjected to the next centrifugation step. Reaching step 6, the supernatant was centrifuged for 70 min at 110.000 xg to separate the exosome pellet which was further PBS-washed and centrifuged at 110.000 xg for additional 70 min to eliminate surplus proteins. The pellet of exosomes was collected and resuspended in 100 µL PBS storage at -80℃ for further work. The centrifugation was carried out using a SW41 swing rotor (Beckman Coulter, Fullerton, CA, USA). A total of 20 µL of exosomes was loaded onto a formvar/carbon-coated grid, negatively stained with 3% aqueous phosphor-tungstic acid for one minute and observed by transmission electron microscopy (HITACHI, H-7650, Japan), micro Bradford protein assay (Biobasic) was used to measure and standardize the protein level of the exosome batch. The dose of injected exosomes was adjusted to 200 µg protein/suspended in 0.2 ml PBS. Additionally, fluorescence-activated cell sorting (FACS) analysis was performed to confirm exosome phenotype. Where a 30 mg of exosomes were incubated for 15 min at room temperature with 10 mL of 4-mm-diameter aldehyde/sulfate latex beads (ThermoFisher Scientific) in a final volume of 100 mL, and then gently shaken for 2 h in 1 mL of PBS. Becton Dickinson's FACS wash was used to wash the exosome-coated beads three times before resuspending them in 500 mL of the solution and Anti-CD80 antibody( abcam, UK, ab215166), PE-labeled anti-CD86 antibody [BU63]—BSA and Azide free (abcam, UK, ab213044,) and anti-MHC-II (SANTA CRUZ BIOTECHNOLOGY, INC, USA (ER-TR3): sc-59318) Santa Cruz Biotechnology, Inc. were then added to 10 mL of coated beads for 1 h. After washing, the samples were analysed using a CYTOMICS FC 500 Flow Cytometer (Beckman Coulter, Brea, CA, USA) and CXP software version 2.2.

### Chronic Pancreatitis Model

CP was induced in rats using L-arginine which was purchased from Sigma Aldrich Co. (USA). Arginine was dissolved in normal saline (20% w/v) and administered to rats [19] as follows: On day 1, the rats received two intraperitoneal injections of arginine solution (250 mg/100 g body weight) spaced one hour apart. On days 4, 7, 10, 13, 16, and 19, the same dose of arginine was administered. Then the rats were sacrificed on day 35.

### Experimental Design

Forty rats were randomly distributed into four groups as follows:

- Control group (group I): Rats received sham injection of normal saline.
- CP group (group II): Rats were subjected to CP protocol.
- Exo treatment group (group III): Rats were exposed to CP just as in group II. Then, they were treated with two tail vein injections of exosomes (200 μg/kg/dose suspended in 0.2 ml PBS). The first injection was on day 21 and second one was on day 28 after tail rubbing by xylene which induces the blood circulation, Rat scarification took place on the 35^th^ day after starting CP protocol [20]
- Exo Prophylaxis group (group IV): Rats were treated with two tail vein injections of exosomes (200 μg/kg/dose suspended in 0.2 ml PBS) 7 and 14 days before starting CP protocol as in group II.

### Blood and Tissue Sampling

At the end of experiment, rats were anesthetized, and then blood samples were collected in dry Wassermann tubes via retro-orbital venous plexus using a fine-walled Pasteur pipette. Samples were centrifuged at 3000 xg for 15 min to separate serum which was divided into aliquots and stored at – 80^ₒ^ C for further analysis.

Under anaesthesia, rats were sacrificed by cervical dislocation. The whole pancreas was instantaneously removed, dried and weighed after being washed with ice-cold isotonic saline. A section of each animal's pancreatic tissue was preserved in 10% formalin for histopathological examination using Hematoxylin and Eosin (H&E) staining, and the remaining tissue was quickly frozen at -80 ◦C for additional biochemical study.

### Assessment of Body Weight

The weight of the rats was measured at the end of experiment by a digital scale.

### Intraperitoneal Glucose Tolerance Test (IPGTT)

The test was performed 24 h before the scarification on 10 h fastened-rats. Rat tail was shaved, and then a small drop of capillary blood was obtained by using sterile lancet and placed on the test strip of blood glucose meter (Bionime Corporation, Taiwan). This measurement was considered the baseline glucose level (t=0). Subsequently, rats received intraperitoneal glucose injection (2 g/kg) of D-glucose (sigma Aldrich Co. USA) that was diluted to a 20% solution using normal saline. Then the blood glucose levels were measured at 30, 60, 90, 120 min following glucose injection.

### Pancreatic Inflammatory Markers

Protein expression levels of IL-10, TNF-α, TGF-β and MMP-9 were assessed in pancreatic tissues by ELISA assay kits supplied by Cloud-Clone Corp, Wuhan, China (Cat. #: SEA056Ra, SEA133Ra, SEA124Ra, SEA553Ra; respectively).

### Pancreatic Enzymes

Serum levels of pancreatic enzymes; lipase and amylase were measured spectrophotometrically using ELISA assay kits provided by Abbexa Ltd, Cambridge, UK (Cat. #: abx492766) and Novus Biologicals, USA (Cat. #: NBP2-68,205) respectively.

### Pancreatic Oxidative Stress Markers

Portions of the pancreatic tissue samples were homogenized in cold PBS to produce 10% homogenate that was used for the spectrophotometric assays of superoxide dismutase (SOD) [21]; catalase [22], glutathione peroxidase (GPx) [23] and malondialdehyde (MDA) levels [24] using commercially available kits provided by Biodiagnostics, Egypt (Cat. #: K335-100, K773-100, K762-100, K739-100) respectively.

### Pancreatic T Cell Subtype

Pancreatic tissues were dissociated with Multi Tissue Dissociation Kit (MiltenyiBiotec). Briefly, the tissue sampels were transferred to serum-free DMEM and the enzyme mix and homogenized at 120 runs for 37 s. After incubation at 37 °C for 20 min under continuous rotation then the samples were dissociated at 168 runs for 37 s and further filtered through a 70 μm cell strainer to exclude extracellular remains. Thereafter the suspension was centrifuged at 300 × g for 6 min. After washing with FACS buffer, 1 × 106 cells per tube were preincubated with 1 μL FcR Blocking Reagent (130–092-575, MiltenyiBiotec). Analysis of T cell subtype was performed using flow cytometry (BD Biosciences, San Jose, CA). The collected cells were and stained with PE-CD3, FITC-CD4 and APC-CD8 (ThermoFisher Scientific), respectively, to detect the percentage of CD3 + T, CD4 + T, and CD8 + T cells.

### Histopathological Study

Pancreatic tissue samples were flushed and fixed in 10% neutral buffered formalin for 72 h. Samples were trimmed and processed in serial grades of ethanol, cleared in xylene; samples were infiltrated and embedded into Paraplast tissue embedding medium. 4 μm thick sections were cut by rotatory microtome and mounted on glass slides. Tissue sections were stained by H&E as a general morphological examination staining method then examined by using light microscope (Leica Microsystems GmbH, Wetzlar, Germany) [25]. The total surface of the slides was scored by two blinded pathologists with expertise in pancreatic pathology. Morphological changes regarding the presence of fibrosis, fat infiltration, inflammation and acinar cell necrosis were defined in 20 randomly selected different fields for each section. Changes were semiquantitavely evaluated using the scoring system reported by Schmidt and his colleagues [26].

### Immunohistochemistry (IHC)

Paraffin sections were mounted on positively charged slides by using avidinbiotin- peroxidase complex (ABC) method. Sections from each group were incubated with monoclonal antibodies for CD4 or CD8 (dilution 1:100) (Catalogue No. A19018 and A22219, respectively, ABclonal technology), then the reagents required for ABC method were added (Vectastain ABC-HRP kit, Vector laboratories). Marker expression was labeled with peroxidase and colored with diaminobenzidine (DAB, Sigma) to detect antigen–antibody complex. Negative controls were included using non-immune serum in place of the primary or secondary antibodies. IHC stained sections were examined via using Olympus microscope (BX-63). The staining intensity was quantitatively scored by the determination of reaction area percent in 10 microscopic fields using image J 1.53t, Wayne Rasband and contributors, National Institutes of Health, USA.

### Statistical Analysis

The mean ± standard deviation (SD) was used to express the Utilizing a one-way analysis of variance, differences between groups were evaluated (ANOVA). For intergroup comparisons, a post hoc Tukey–Kramer test was then run. The program Prism 2007 (GraphPad software, Inc., San Diego, USA), version 5.01, was utilized. At p < 0.05, a significant difference was declared.

## RESULTS

### Identification of BMDCs

#### Inverted Microscope

**Fig. 2:**
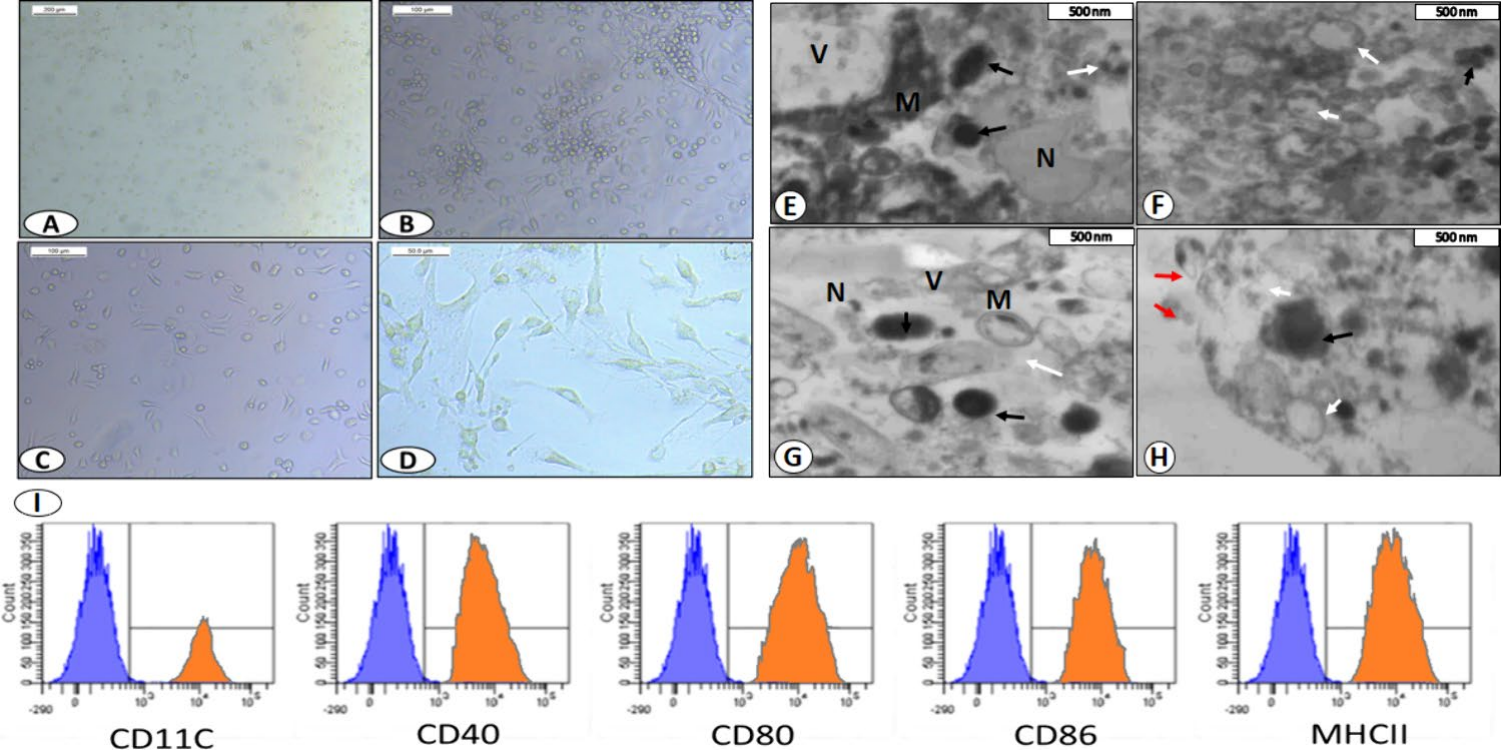
**A**-**D** Tracking the continuous morphological changes of BMDCs at different interval times; cells after 1 day incubation (A), cells after 3 days incubation (B), cells after 6 days incubation (C), cells after 12 days incubation (D). **E**–**H** Ultrastructural analysis of dendritic cells by transmission electron microscopy; M: Mitochondria, N: Nucleus, V: Vacuole, White arrows: Vesicles budding off the nuclear envelope with cytoplasmic granules migrating to the plasma membrane, Red arrows: Vesicles Released out from the plasma membrane, Black arrow Electron-dense cytoplasmic granules. Scale bars are as shown. **I** FACS analysis for cultured BMDCS.

After 24 h of incubation with rrGM-CSF, inverted phase-contrast microscopy showed that some of the cells adhered to the plate and some other were suspended in the culture medium. On day 3, the formation of colonies could be recognized. After 6 days of incubation, both the size and the number of the suspended cells considerably increased. Moreover, few cells with dendritic protrusions were first noticed, while the number of adhering cells decreased over time. After adding IL-4, the suspended cells with dendritic protrusions increased and showed gradual elongation of the protrusions. The colonies dispersed 48 h after the addition of TNF-α and the cells were uniformly distributed all over the medium and mature dendrites could be seen (Fig. 2A-D).

#### Electron Microscope

Ultrastructural analysis of BMDCs by transmission electron microscopy showed many mitochondria, nuclei and vacuoles. Vesicles were seen budding off the nuclear envelope with cytoplasmic granules migrating to the plasma membrane (Fig. 2E-H).

#### Flow Cytometric Detection of BMDCs Phenotypes

FACS analysis of the cultured BMDCs revealed that they were positive for CD11C (56%), CD40 (90%), CD80 (88.5%), CD86 (82.4%) and MHCII (98.5%) as shown in Fig. 2I.

### Identification of BMDCs Derived Exosomes

#### Electron Microscope

**Fig. 3:**
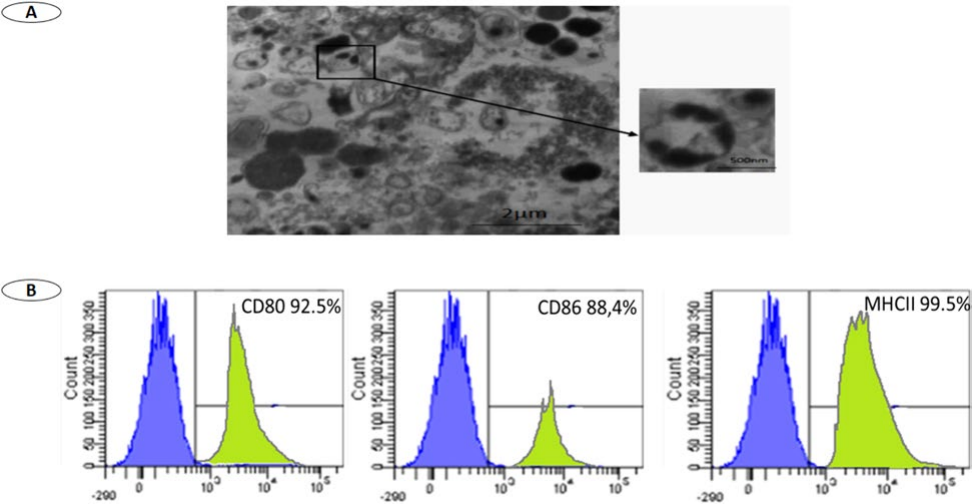
**A** Transmission electron microscope analysis of BMDCs exosomes; Right photo: highly magnified single exosome (size is less than 70 nm) illustrated at scale bar of 500 nm. Left photo: exosomes illustrated at scale bar of 2 µm. **B** FACS analysis for BMDCs derived exosomes.

Transmission electron microscopy ultrastructure analysis of BMDCs derived exosomes revealed their characteristic spheroid double-membrane bound morphology with a diameter of 40–100 nm (Fig. 3A).

#### Flow Cytometric Detection of BMDCs Derived Exosomes Phenotypes

BMDCs derived exosomes showed positive expression of the following cell surface markers; CD80 (92.5%), CD86 (88.4%) and MHC11 (99.5%) as shown in Fig. 3B.

### Effect of BMDCs Exosomes on Body Weight in L-arginine-induced CP

**Fig. 4:**
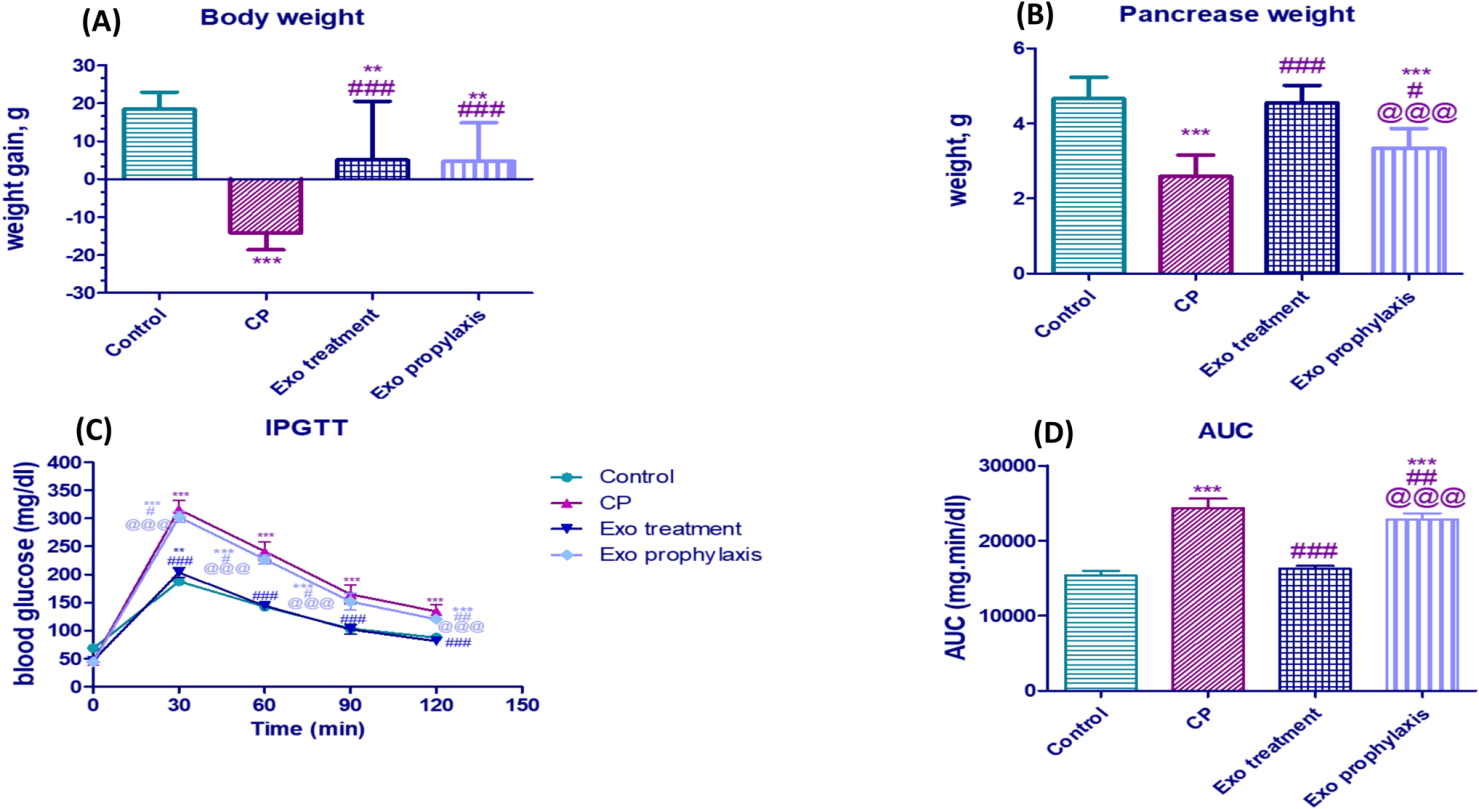
Effect of BMDCs exosomes on CP-induced changes; weight gain (**A**), pancreas weight (**B**) IPGTT (**C**) and AUC (**D**). Each bar with vertical line represents mean ± SD (10 rats/group). *versus control, # versus CP, @versus Exo treatment, 1 symbol denotes *p* < 0.05, 2 symbols *p* < 0.01, 3 symbols *p* < 0.001 and 4 symbols *p* < 0.0001, using one-way ANOVA followed by Tukey’s post hoc test.

Because of the cachexia effect of chronic pancreatitis, body weight of the rats in CP group showed a significant reduction that reached 14.17% of the initial weight. On the other hand, weight gain amounting to 18.50% in the control group, 5% in the Exo treatment group and 4.7% in the Exo prophylaxis was recorded (Fig. 4A).

### Effect of BMDCs Exosomes on Pancreatic Weight in L-arginine- induced CP

Compared to CP group, both Exo treatment and Exo prophylaxis groups showed significant weight improvement of the CP-induced pancreatic atrophy approaching 1.7 fold (*P* < 0.001) and 1.2 fold (*P* < 0.05) respectively (Fig. 4B).

### Effect of BMDCs Exosomes on IPGTT in L-arginine-induced CP

The blood glucose levels in CP group were significantly higher than those in the control group. Compared to CP group, Exo treatment group showed significant decrease at 30, 60, 90 and 120 min with statistical difference of *P* < 0.001, while the Exo prophylaxis showed a significant decrease at 30, 60, 90 min (*P* < 0.05) and 120 min (*P* < 0.01) (Fig. 4C), the results were expressed using AUC values as well (Fig. 4D).

### Effect of BMDCs Exosomes on Pancreatic Inflammatory Markers in L-arginine- induced CP

**Fig. 5:**
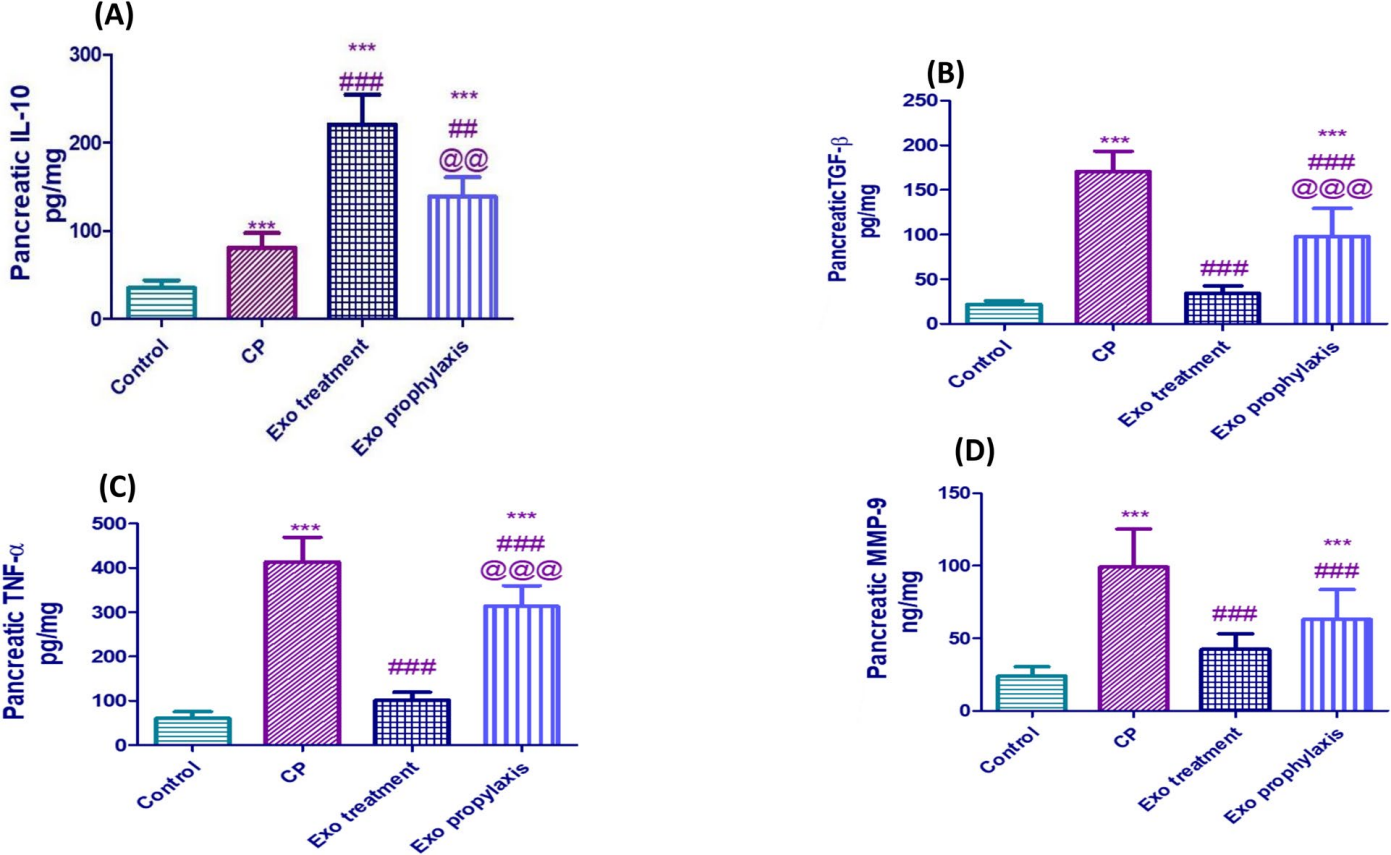
Effect of BMDCs exosomes on CP-induced changes regarding pancreatic IL-10 (**A**), TGF-β (**B**) TNF-α (**C**) and MMP-9 (**D**). Each bar with vertical line represents mean ± SD (10 rats/group). *versus control, # versus CP, @versus Exo treatment, 1 symbol denotes *p* < 0.05, 2 symbols *p* < 0.01, 3 symbols *p* < 0.001 and 4 symbols p < 0.0001, using one-way ANOVA followed by Tukey’s post hoc test.

The pancreatic protein expression levels of IL-10, TNF-α, TGF-β and MMP-9 are crucial to assess progression of inflammation in pancreas. As shown in Fig. 5A, the rats in the CP group showed a marked elevation of pancreatic IL-10 level compared to control (2.3 fold, *p* < 0.001). Such effect was relieved by the administration of exosomes which caused 2.75- and 1.7-fold increase in Exo treatment and Exo prophylaxis groups, respectively, compared to CP group. CP rats showed a 6.7, 8, 4.1-fold increase of TNF-α, TGF-β and MMP-9 pancreatic levels compared to the control level (*p* < 0.001). The tissue expression levels of TNF-α, TGF-β and MMP-9 in Exo treatment group significantly decreased to 75.5%, 80% and 58% of that of the CP group level (*p* < 0.001), while the rats in Exo prophylaxis revealed 26.6%, 42.4% and 36.4% decline in pancreatic TNF-α, TGF-β and MMP-9 levels; respectively compared to the CP group (Fig. 5B-D).

### Effect of BMDCs Exosomes on Pancreatic Enzymes Levels in L-arginine- induced CP

**Fig. 6:**
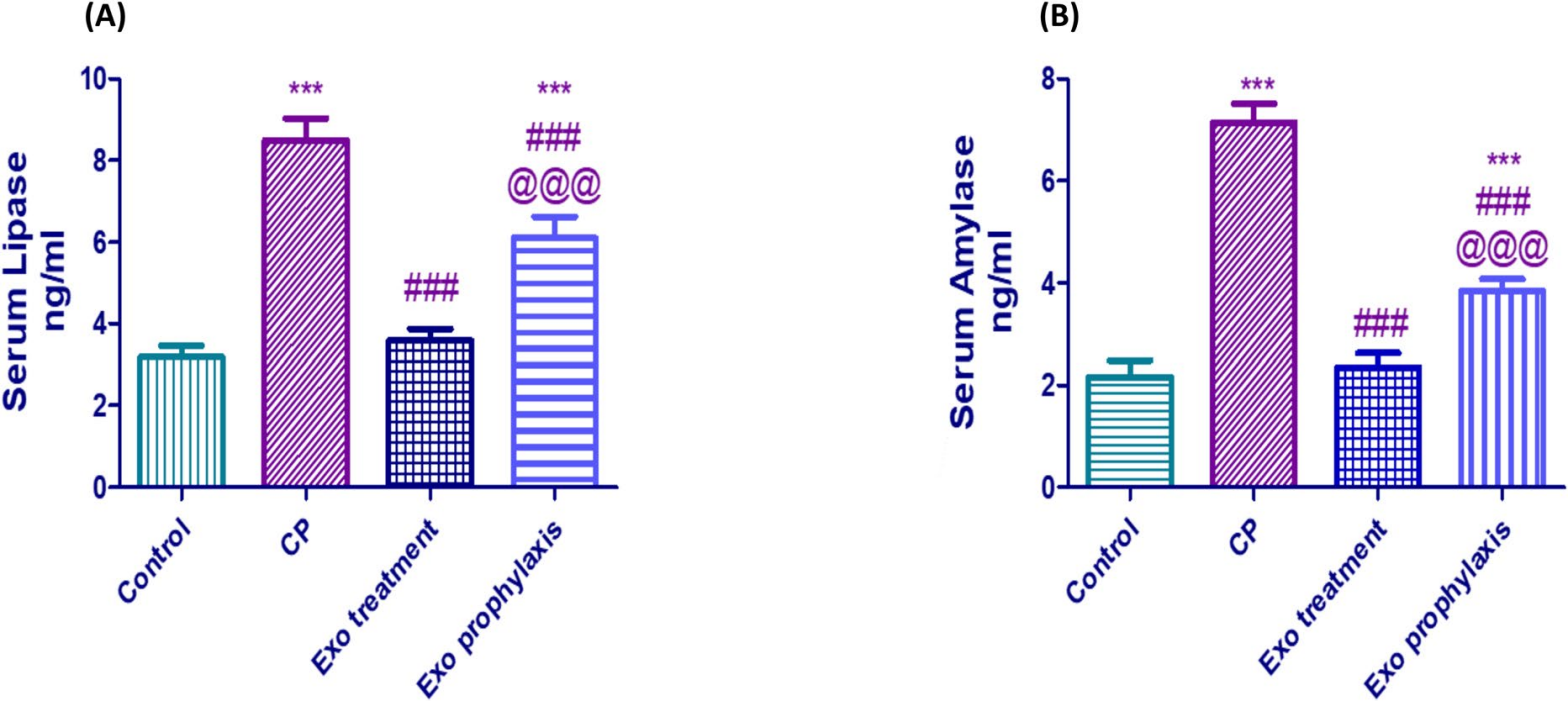
Effect of BMDCs on exosomes CP-induced changes regarding the enzyme levels of lipase (**A**) and amylase (**B**). Each bar with vertical line represents mean ± SD (10 rats/group). *versus control, # versus CP, @versus Exo treatment, 1 symbol denotes *p* < 0.05, 2 symbols *p* < 0.01, 3 symbols *p* < 0.001 and 4 symbols *p* < 0.0001, using one-way ANOVA followed by Tukey’s post hoc test.

Both serum amylase and lipase protein expressions were significantly elevated in CP group to 3.5 and 2.8 fold compared to control group (*p* < 0.001), but this elevation was hampered in Exo treatment group to 67% and 58% of the activities in CP group (*p* < 0.001) and in Exo-prophylaxis group by 47% and 30% compared to CP group (*p* < 0.001) as shown in Fig. 6A &B.

### Effect of BMDCs Exosomes on Pancreatic Oxidative Stress Markers in L-arginine- induced CP

**Fig. 7:**
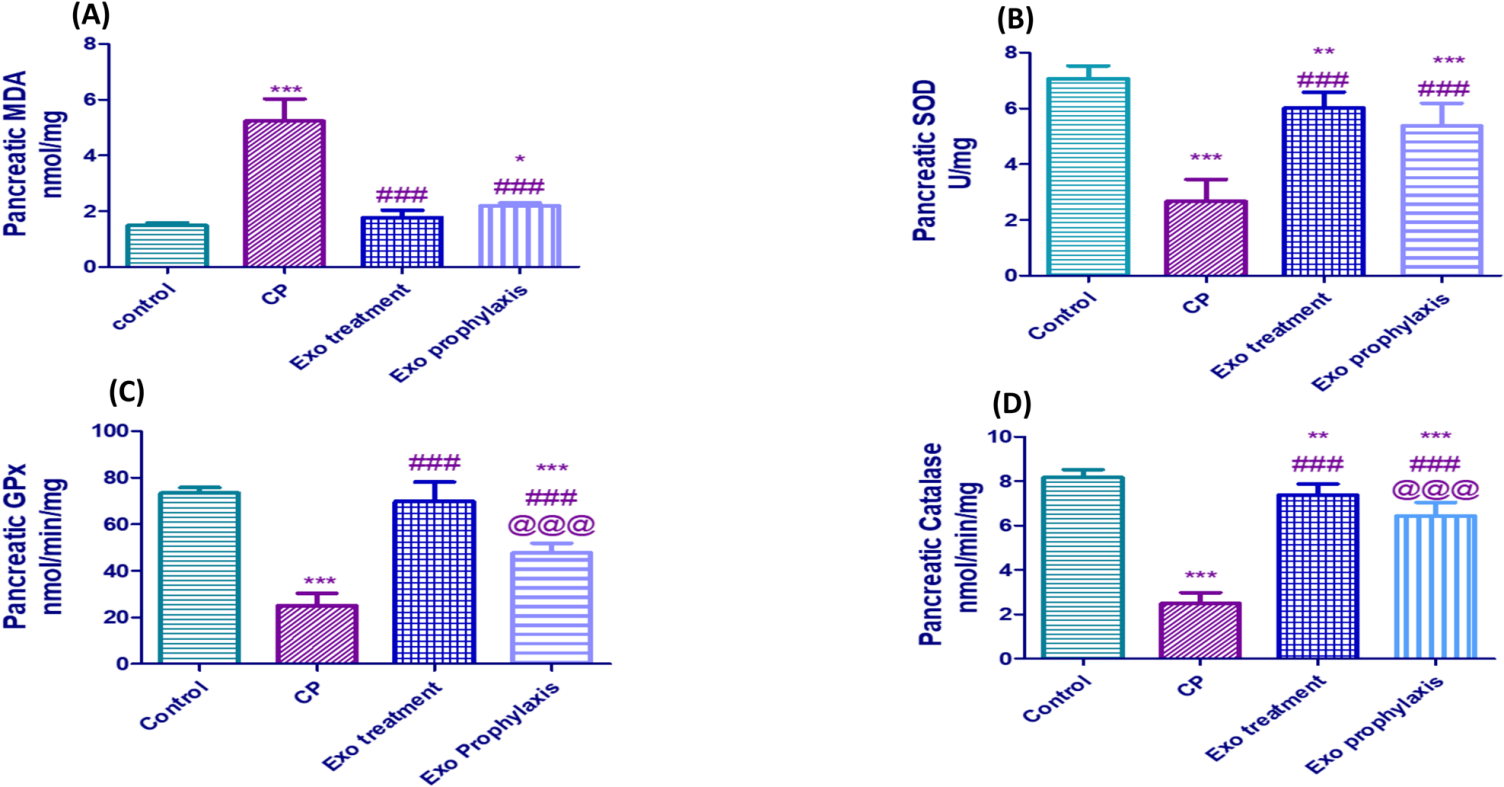
Effect of BMDCs exosomes on CP-induced changes regarding the pancreatic oxidative stress markers; MDA (**A**), SOD (**B**), GPx (**C**) and Catalase (**D**). Each bar with vertical line represents mean ± SD (10 rats/group). *versus control, # versus CP, @versus Exo treatment, 1 symbol denotes *p* < 0.05, 2 symbols *p* < 0.01, 3 symbols *p* < 0.001 and 4 symbols *p* < 0.0001, using one-way ANOVA followed by Tukey’s post hoc test.

CP induced ROS-mediated cell membrane damage as it dramatically levelled up MDA pancreatic concentration reaching 3.7 fold that of the control group (*p* < 0.001), whereas Exo treatment and Exo prophylaxis caused a 67.3% and 57.7% decrease considering the CP group (*p* < 0.001). CP showed significant decrease of pancreatic SOD, catalase and GPx activities by 63%, 69.5% and 66% (*p* < 0.001) compared with the control group. Treatment of CP rats with exosomes caused a significant increase in pancreatic SOD, catalase and GPx activities by 2.3, 3, and 2.8 fold (*p* < 0.001) considering the CP group. Meanwhile, using exosomes before CP induction significantly increased pancreatic SOD, catalase and GPx activities to 2, 2.6, and 1.9 fold those of CP group (*p* < 0.001) (Fig. 7).

### Effect of BMDCs Exosomes on Pancreatic T Cell Subtype in L-arginine induced CP

**Fig. 8:**
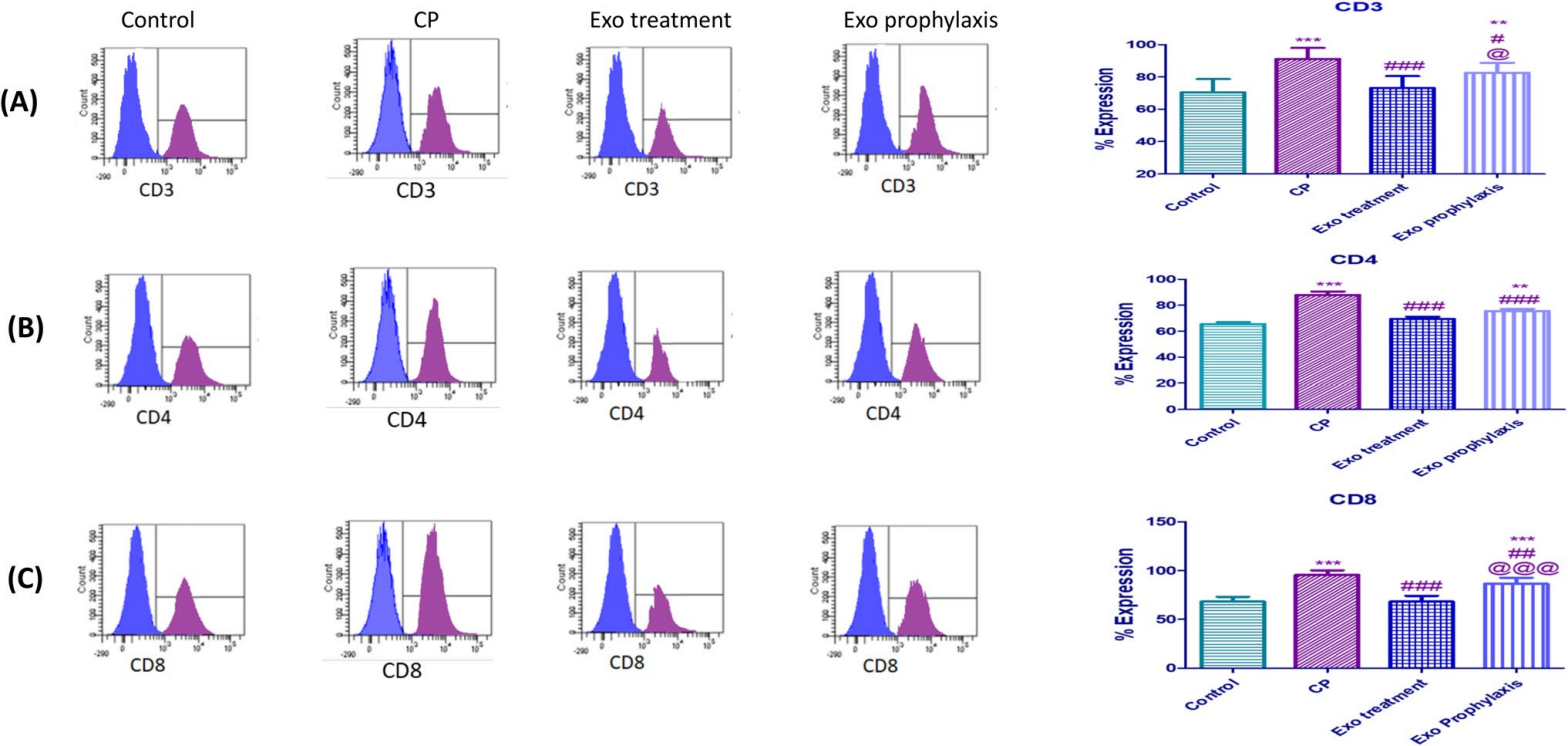
Effect of BMDCs exosomes on CP-induced changes regarding the pancreatic T cell subsets; CD3 (**A**), CD4 (**B**) and CD8 (**C**). Each bar with vertical line represents mean ± SD (10 rats/group). *versus control, # versus CP, @versus Exo treatment, 1 symbol denotes *p* < 0.05, 2 symbols *p* < 0.01, 3 symbols *p* < 0.001 and 4 symbols *p* < 0.0001, using one-way ANOVA followed by Tukey’s post hoc test.

As shown by the flowcytogram in Fig. 8, the expression of CD3, CD4 and CD8 positive T cells in CP group increased by 70.3%, 65.5% and 68.3% respectively compared to control group but these levels decreased significantly in both Exo treatment and prophylaxis groups when they are compared to the levels of CP group.

### Histopathological Examination

#### Microscopic Examination of Different Rat Pancreatic Tissue Samples

**Fig. 9:**
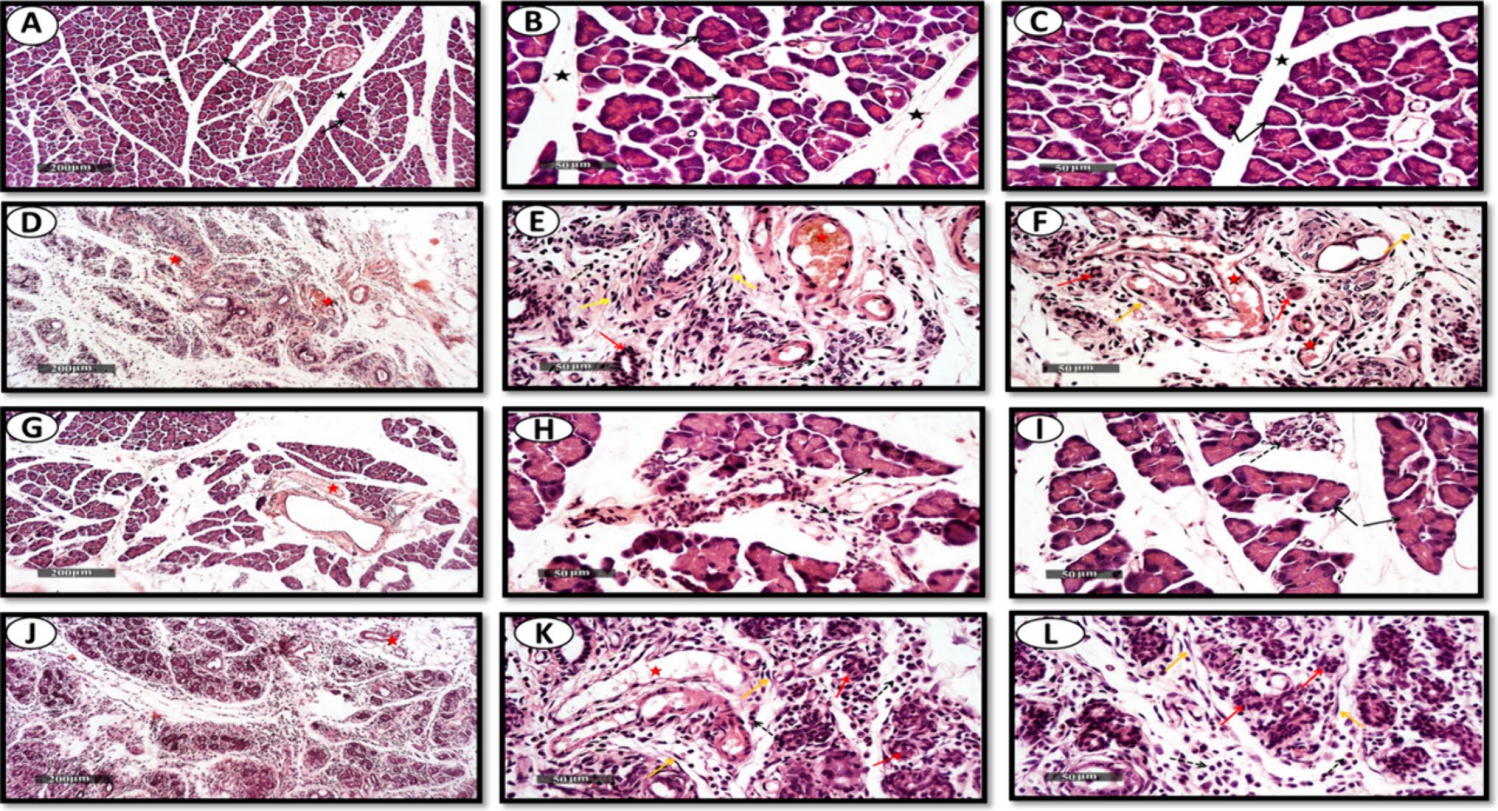
Effect of BMDCs exosomes on CP-induced pancreatic histopathological changes. Representative photomicrographs illustrating H & E staining of pancreas from control group (**A**, **B** and **C**), CP group (**D**, **E** and **F**), Exo treatment group (**G**, **H** and **I**) and Exo prophylaxis group (**J**, **K** and **L**). Black arrows, intact pancreatic acini; black stars, normal lobules separated by connective tissue septa; red arrows, degenerative pancreatic acini; dashed arrows; inflammatory cells infiltrates; yellow arrows, collagen fibres contents; red stars, congested blood vessels.

Control samples demonstrated normal histological features of pancreatic parenchyma including exocrine and endocrine compartments with almost apparent intact well organized pancreatic acini showing intact subcellular details with abundant zymogenic granules (arrows) in different lobules separated by delicate interlobular connective tissue septa (black star) with normal vasculatures and minimal inflammatory cells infiltrates (Fig. 9A-C).While the tissues separated from the CP group showed diffused severe degenerative and necrotic changes with significant loss and atrophy of pancreatic acini (red arrows) accompanied with severe mononuclear inflammatory cells infiltrates (dashed arrow), abundant fibroblastic activity with significant higher collagen fibers contents (yellow arrow) and many dilated and congested blood vessels (red star) as shown in (Fig. 9D-F). Exo treatment group samples demonstrated moderate higher protective efficacy against CP and significantly higher densities of apparent intact well-organized pancreatic acini (black arrow). Significant reduction of interlobular spaces was observed with moderate persistence of interlobular and perivascular focal inflammatory cells infiltrates (dashed arrow), mild congested blood vessels (red star) as well as collagen fibers formation with minimal fibroblastic activity as shown in (Fig. 9G- I). On the other hand, the samples separated from Exo prophylaxis group showed less improvement as shown in Fig. 9J-l.

#### Histopathology Score

**Fig. 10:**
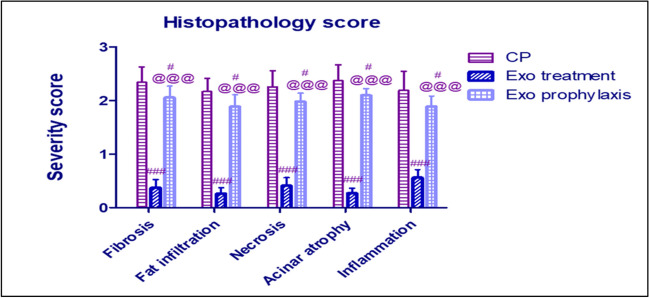
Effect of BMDCs exosomes on CP-induced changes in pancreatic histopathology scores. Each bar with vertical line represents mean ± SD (10 rats/group). *versus control, # versus CP, @versus Exo treatment, 1 symbol denotes *p* < 0.05, 2 symbols *p* < 0.01, 3 symbols *p* < 0.001 and 4 symbols *p* < 0.0001, using one-way ANOVA followed by Tukey’s post hoc test.

CP group has shown significantly high severity scores for fibrosis, fat infiltration, necrosis, acinar atrophy and inflammation, while the severity scores of such changes were significantly better in Exo treatment and Exo prophylaxis groups compared to those of CP group (Fig. 10).

#### Effect of BMDCs Exosomes on Pancreatic Protein Expression of CD4 and CD8 in L-arginine induced CP

**Fig. 11:**
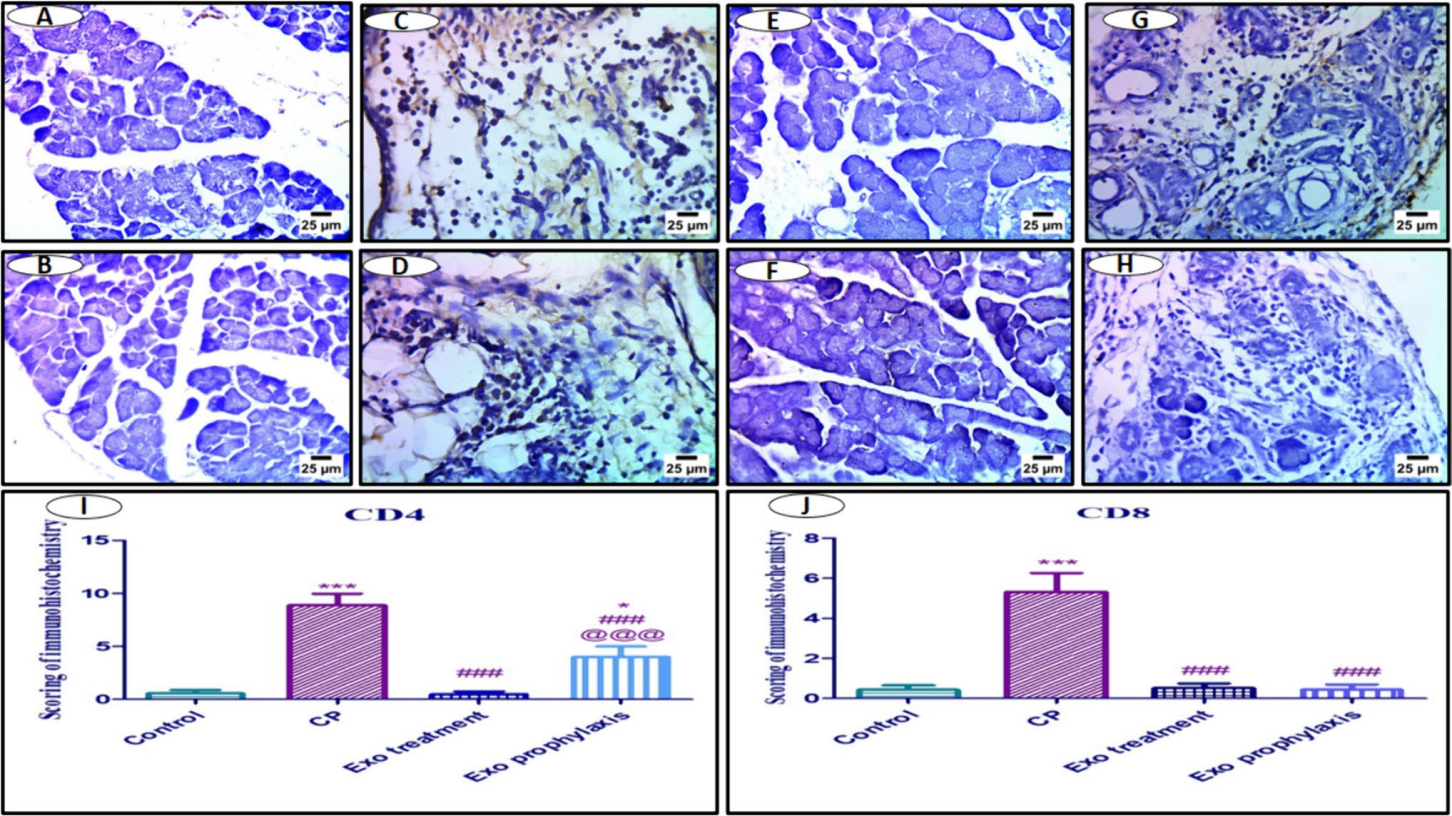
Representative photomicrographs illustrating (IHC-Peroxidase-DAB) staining of pancreatic tissue samples from different groups. Control group (**A** & **B**) showing negative expression for CD4 and CD8, respectively, CP group (**C** & **D**) showing positive expression for CD4 and CD8 (arrow), respectively, Exo treatment group (**E** & **F**) and Exo prophylaxis group (**G** & **H**) showing reduced expression for CD4 and CD8, respectively. Each bar with vertical line in the bar charts (**I** & **J**) represents mean ± SD (10 rats/group). *versus control, # versus CP, @versus Exo treatment, 1 symbol denotes *p* < 0.05, 2 symbols *p* < 0.01, 3 symbols *p* < 0.001 and 4 symbols *p* < 0.0001, using one-way ANOVA followed by Tukey’s post hoc test.

Immunohistochemical examination showed significantly higher expression of CD4 and CD8 by 17 folds and 11.5 folds, respectively, in the pancreatic tissues of CP group compared to the control group. Both Exo treatment and Exo prophylaxis groups demonstrated a significant decrease of the pancreatic expression of CD4 and CD8 compared to CP group (Fig. 11).

## DISCUSSION

Chronic pancreatitis is a progressive fibro-inflammatory disorder impairing permanently the exocrine and endocrine functions of the pancreas, often leading to unrelenting chronic pain and negative effects on the patients’ wellbeing. Furthermore, CP is associated with high mortality rates and is identified as the strongest risk factor for pancreatic cancer [6]. Therefore, efficient treatments beyond the commonly used supportive and symptomatic therapy have to be explored. Regenerative medicine including the use of exosomes is attracting nowadays more attention [27].

In the current study, we tracked various pathological, histological and biochemical changes related to CP, and to mimic the course of CP-induced disorders in humans we used arginine-induced CP rodent model [28]. L-arginine is a substrate for the enzyme nitric oxide synthase (NOS), which produces nitric oxide (NO), aids in the urea cycle, and eliminates ammonia from the body [29]. The majority of endogenous L-arginine is produced in the kidneys from citrulline. In newborns, the synthesis could not be enough [30], so L-arginine is categorized as a conditionally essential amino acid. L-arginine supplementation in the daily recommended doses has been suggested to treat cardiovascular diseases, enhance sexual performance [31–33], reduce obesity and metabolic syndrome, improve muscle performance of athletes and bodybuilders, and promote wound healing [34]. However, long-term L-arginine supplementation increases formation of NO and ROS that damage DNA, proteins and lipids, inducing apoptosis in different body organs and soft tissues, specifically the pancreas, leading to chronic inflammation and β-cell destruction [35, 36]. Furthermore, arginine binds to certain G-protein coupling receptors in pancreas causing their imbalanced activation, which contributes to the development of pancreatitis [37].

The present study revealed the adverse effects of CP on body weight, pancreas weight, IPGTT, pancreatic enzymes, pancreatic inflammatory and oxidative stress markers in addition to pancreatic T cell subtypes in rats. The study's major finding is that BMDCs exosomes represent a promising candidate for prophylactic and therapeutic purposes regarding CP-induced disorders.

DC-derived exosomes were selected in our study for many reasons. Besides their sustained stability following freezing, they display such a molecular composition that fortifies their immunoregularory properties and bio functionality suggesting their effective therapeutic potential. Whereas, BMDCs exosomes outer membrane is dense in MHC peptide complexes that can be transmitted to surrounding antigen presenting cells. BMDCs have as well a high potential to trigger natural killer cells [38].

Our study showed that CP is associated with body weight loss. The results were in line with those of the previous study of Bachmann et al. [39]. Weight loss is mainly referred to the CP-induced pancreatic acinar cells destruction and exocrine function impairment. Physiologically, the pancreas secretes digestive enzymes into the duodenum facilitating the breakdown of micronutrients and macronutrients. Therefore, untreated CP is characterised by maldigestion and malabsorbtion which is exacerbated by precipitation of bile acids resulting in steatorrhea; loss of essential fatty acids and fat-soluble vitamins in stool, all of which lead to weight loss. Moreover, CP patients frequently eat too little because of the abdominal pain linked to food consumption, furthermore, Gastroparesis which has been seen in up to 44% of CP cases, may trigger distressing gastrointestinal symptoms including nausea and vomiting, which additionally decreases oral food intake and worsens clinical outcomes. Because of the progressive inflammation, cases with CP frequently exhibit a higher basal metabolic rate of about 110% of the baseline (35 kcal/kg/24 h). This accounts for the initial 10% to 20% loss of body weight, along with poor oral food consumption [40].

Our results indicated that body weight gain was recovered in both Exo treatment and Exo prophylaxis groups. This may be attributed to the immunoregulatory properties of exosomes generated from DCs especially those treated by anti-inflammatory molecules such as IL10 and IL4. Such exosomes represent therapeutic option against different immune and inflammatory disorders and probably improve the CP affected pancreatic function [41]. A study demonstrated that intravenous administration of umbilical cord mesenchymal stem cells (UC-MSC) exosomes to mice with steatohepatitis reduced body weight loss and liver damage brought on by methionine–choline-deficient dietShi, Yang, Wang, Wu, Zheng, Tang, Gao and Niu [42]. Furthermore, UC-MSC exosomes restored the downregulated Peroxisomal proliferator-activated receptor (PPARα) and reduced the inflammatory cytokines levels in the liver tissues. Similarly, mesenchymal stem cells exosomes showed effective restoration of total body weight in chemotherapy-treated mice via immunoregulatory mechanism including miR-145-5p [43].

Rats exposed to CP showed statistically smaller pancreas compared to controls, which was supported by pancreatic histopathology findings. The high severity score of acini atrophy and necrosis are hallmarks of pancreas weight reduction [28, 44, 45]. The present study revealed that BMDCs exosomes in both Exo treatment and Exo prophylaxis groups suppressed oxidative stress and attenuated necrosis contributing to tissue repair and cell regeneration and thereby the maintenance of normal pancreas weight. These results are in agreement with those of several previous studies Wang, Tian, Tang, Rui, Tian, Ma, Ma, Xu, Lu and Wang [20, 46, 47].

Pancreatic endocrine dysfunction in CP, which occurs secondary to fibrotic replacement of Langerhans islets was evidenced in our study and other previous studies by the impaired glucose intolerance, which is collectively called pancreatogenic (Type 3c) diabetes [28, 45]. CP's underlying pathophysiology of diabetes is mostly dependent on the disrupted insulin secreting capacity of the pancreatic islets. However, insulin resistance is suggested as well to contribute to the development of diabetes in CP. In opposing studies, episodes of fatal hypoglycaemia were reported, which probably go to the poor ability of alpha cells to produce glucagon to act as a counterbalance. The chronic inflammation induced by arginine administration disrupts not only the vascular microenvironment, but also the antioxidant status of the endocrine pancreas which subsequently impair glucose homeostasis [28]. The islets depend on their dense perfectly-organized microvasucular network to adequately respond to changes in plasma glucose levels. The homeostasis and the function of the islets microvasulature are maintained mainly by the controlled expression of vascular endothelial growth factor (VEGF) [48]. Preventing the gene expression of the signal transducer and activator transcription 3 (STAT-3), a transcription factor regulating the expression of VEGF was demonstrated to cause pancreatic hypovascularisation which was associated with glucose intolerance in mice [49]. Altered angiogenesis was detected by a bioinformatic analysis in the inflamed pancreas of CP clinical cases. Moreover, disrupted angiogenesis in the exocrine pancreas was suggested to cause the death of neighbouring beta cells through TGFβ1-triggered beta-cell epithelial–mesenchymal transition [50].

Regarding the antioxidant status, several experimental and clinical studies implicated the redox imbalance in the pathogenesis of CP [51]. Enhanced oxidative stress was reported as well by the present study in CP group that showed increased MDA levels and diminished activities of pancreatic antioxidant enzymes; SOD, catalase and GPx. CP patients show low antioxidant capacity probably due to the increased consumption of antioxidants to combat oxidative stress in addition to the impaired absorption leading to dietary deficiency. The excessive production of ROSs cause lipid peroxidation, DNA damage, and protein denaturation leading to cellular pancreatic injury [51]. However, the pancreatic islets are more prone to oxidative stress induced damage. They are scattered all over the pancreas and represent only 2% of its tissues, in contrast to the acinar cells that constitute more than 80% of the pancreatic tissues. Furthermore, the B-cells exhibit very weak antioxidant defence system compared to other pancreatic cells. That why, they are drastically affected when the pancreas is subjected to oxidative stress, which is reflected on the ability to produce insulin and to maintain glucose homeostasis [28, 52].

Interestingly, the pancreatic endocrine function was relatively maintained in both Exo treatment and Exo prophylaxis groups that showed significantly lower blood glucose levels compared to CP group. Based on previous reports, exosomes are beneficial in treating diabetes mellitus. They have direct protective effect on the pancreatic β cells promoting their regeneration and insulin secretion. The mechanistic process involves enhancing the expression of Pdx-1, TGF-β, Smad2, and Smad3 [53, 54]. Thanks to their antiapoptotic activity, exosomes probably relieved the endoplasmic reticulum stress in β cells helping them to retain the capacity to secrete insulin [55]. Moreover, exosomes are speculated to aid the pancreatic cell repair and regeneration via protecting the pancreatic vasculature [41]. Exosomes showed positive effects on maintaining stable angiogenesis in various disease models through transmitting diverse biomolecules like microRNAs and long non-coding RNAs to recipient cells and upregulating angiogenesis-related molecules; VEGFA and VEGFR-2 [56–58]. Indirectly, exosomes promote the glucose transporters GLUT1 and GLUT4 enhancing glucose uptake by the cells [59]. Additionally, BMDCs exosomes in the present study abrogated the exhaustion of the pancreatic antioxidant system and restored effectively the normal pancreatic antioxidant activity in both Exo treatment and Exo prophylaxis groups. Matching with the present study’s data, DOX-induced oxidative stress could be mitigated by exosomes. This had to do with the antioxidant proteins and miRNAs that exosomes transmit. According to a proteomics investigation, more than 70 proteins were implicated in redox activities were found in exosomes from human right cardiac atrial appendage tissue including SOD2, thrombospondin 1, and collagen 1A1 [60].

The positive effects of BMDCs exosomes on the pancreatic function and structure are further reflected by their potential to reduce the significant upsurge in serum amylase and lipase levels compared to CP group. Exosomes contribute to the repair and regeneration of insulted pancreatic tissues through inhibiting the activation of nuclear factor kappa B (NF-κB) signalling and reducing the infiltration of inflammatory cells. Following pancreatic injury, an inflammatory cascade is triggered by NF-κB, which consequently activates a panel of pro-inflammatory cytokines including TNF-α, IL-6, and IL-18. The activated cytokines transmit signals that initiate the infiltration of neutrophils and lymphocytes that activate the pancreatic stellate cells (PSCs), which proliferate and transform into myofibroblasts and promote collagen synthesis. Along with the further secretion of local cytokines and excessive deposition of collagen, the fibrotic regions expand in the pancreas replacing the glandular parenchyma, thus promoting the development of CP [37, 41]. This could be evidenced in our experimental model of pancreatitis as well as in the previous study that reported an overexpression of TNF-α in both acinar and infiltrating cells after the induction of CP which was significantly correlated with the degree of fibrosis [61]. Exosomes inhibit NF-κB activation probably through inhibiting the TLR4/MyD88/NF-κB signaling pathway, which thereby explain the significant decrease of the pancreatic expression of TNF-α in both Exo groups, contributing to the reduction of the infiltration of inflammatory cells and thus to the repair of the pancreatic cells [41]. In harmony to the current results, exosomes obtained from BMDCs treated with IL‐10 were reported to be superior to those derived from immature DCs in terms of their ability to attenuate increased expression of TNF‐α, IL‐2 and interferon-gamma (IFN‐γ) in colon tissues, suppressing more effectively chemically induced colitis inflammation in rats [62].

On the other hand, we observed that BMDCs exosomes augmented the elevated level of the anti-inflammatory cytokine IL-10 and reduced the overexpressed TGF-β. Upon pancreatic insult, the endogenous production of IL-10 is enhanced in a compensatory process to lessen the severity of the inflammation. IL-10 has direct antifibrotic effect reducing collagen deposition in the pancreas. It does not only supress collage synthesis, but also upregulates the expression of collagenase enzyme [63]. Additionally, IL-10 prevents ATP depletion and pancreatic acinar cell necrosis by increasing the expression of the anti-apoptotic B-cell lymphoma 2 (Bcl-2), which blocks the apoptotic pathway by modulating mitochondrial cytochrome-c release [64]. Indirectly, IL-10 can protect both acinar and interstitial pancreatic cells against fibrotic remodelling, through modulating the production of TGF-β, which is the most potent activator of collagen synthesis by PSCs [65]. In particular, TGF-β induces the connective tissue growth factor, leading to the production of fibronectin, proteoglycan and collagen and the excessive disposition of extracellular matrix (ECM) molecules. Further, TGF-β exerts a potent immunosuppressive effect inhibiting the immune function during the process of chronic inflammation [66]. Along the course of CP, the effect of TGF-β seems to be more pronounced, probably due to the continued secretion of stimulatory cytokines, and could not be counterbalanced by IL-10 [63]. We assume that BMDCs exosomes managed to bring the balance in favour of IL-10. The protective role of exosomes extended to reduce the significantly overexpressed pancreatic MMP-9. Elevated MMP-9 contribute to the development of CP via degrading type IV collagens in normal basement membrane, mediating a bad environment for many cells, and further activating PSCs [67, 68].

The pathogenesis of CP, including the destruction of pancreatic tissues involves mainly cell-mediated cytotoxicity [69–71]. This can be evidenced in the current study and other supporting studies by the dysregulation of immune cells, manifested by the significant increase of CD3^+^, CD4^+^ and CD8^+^ T-cell infiltrates observed in the lesions of CP group compared with control [66, 72]. The infiltration of perforin-expressing CD8^+^ T-cells is considered to be a major contributor in CP pathology and disease severity [69, 73] and associated with the apoptotic death of acinar cells and their replacement with fibrotic masses [74]. Moreover, IL-22 which is produced by CD4^+^ T-cells promotes PSCs activation and ECM deposition which leads to enhanced tissue fibrosis [75].The immune imbalance in T-lymphocytes was corrected in both protective and therapeutic Exo groups. It has been demonstrated that exosomes contain a wide range of immune-related molecules, such as MHC class I and II molecules, lysosome-associated membrane proteins and costimulatory molecules like CD86, therefore they, like their DCs, can directly and indirectly, stimulate immune responses. DCs exosomes can promote the polarization of macrophages and impede the proliferation of natural killer cells and inflammatory CD4 + T, CD8 + T cells [76]. A previous study has demonstrated that DCs exosomes can significantly upregulate the infiltration of regulatory T cells (Treg) and that the activated Treg cells regulate in turn the polarization of macrophages, eliciting an earlier shift in the macrophage subset from inflammatory M1 to reparative M2 macrophages [77]. Treg cells could stimulate macrophages producing IL-10 by secreting IL-13 [78]. Considering the secretion of IL-10 is one of the characteristic features of M2-like type macrophages [76] We assume that this modulation in the immune environment within the inflamed pancreas resulted in better healing.

In conclusion, the present study demonstrated that BMDCs derived exosomes elicited protective and therapeutic effects in the chronic setting of pancreatitis in rats. Their effects were likely mediated through the recovery of pancreatic structure and function after arginine-induced injury. Thereby, the use of BMDCs derived exosomes provides a novel promising strategy to manage CP.

## Data Availability

No datasets were generated or analysed during the current study.
